# Rapid visual adaptation persists across saccades

**DOI:** 10.1016/j.isci.2021.102986

**Published:** 2021-08-16

**Authors:** Carolin Hübner, Alexander C. Schütz

**Affiliations:** 1 Allgemeine und Biologische Psychologie, Philipps-Universität Marburg, 35037 Marburg, Germany; 2 Institut für Psychologie, Humboldt-Universität zu Berlin, 12489 Berlin, Germany; 3 Center for Mind, Brain and Behavior, Philipps-Universität Marburg, 35037 Marburg, Germany

**Keywords:** Behavioral neuroscience, Clinical neuroscience, Sensory neuroscience, Techniques in neuroscience

## Abstract

Neurons in the visual cortex quickly adapt to constant input, which should lead to perceptual fading within few tens of milliseconds. However, perceptual fading is rarely observed in everyday perception, possibly because eye movements refresh retinal input. Recently, it has been suggested that amplitudes of large saccadic eye movements are scaled to maximally decorrelate presaccadic and postsaccadic inputs and thus to annul perceptual fading. However, this argument builds on the assumption that adaptation within naturally brief fixation durations is strong enough to survive any visually disruptive saccade and affect perception. We tested this assumption by measuring the effect of luminance adaptation on postsaccadic contrast perception. We found that postsaccadic contrast perception was affected by presaccadic luminance adaptation during brief periods of fixation. This adaptation effect emerges within 100 milliseconds and persists over seconds. These results indicate that adaptation during natural fixation periods can affect perception even after visually disruptive saccades.

## Highlights

• Retinotopic luminance adaptation can affect postsaccadic contrast perception
• Adaptation builds up rapidly within the natural duration of fixations
• Adaptation persists over long delays of more than one second

## Graphical abstract

**Figure:**
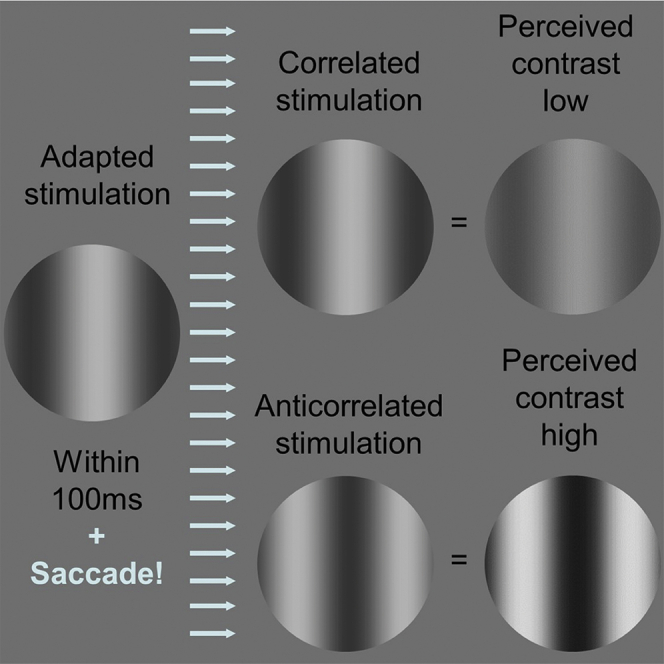

## Introduction

Humans frequently accelerate their eyes to extremely high velocities (Bahill et al., 1975; Baloh et al., 1975) even though such saccadic eye movements disrupt and distort visual processing (for a review see Binda and Morrone, 2018). Nevertheless, humans make saccades about 2–4 times per second (e.g. Otero-Millan et al., 2013). What is the advantage of this behavior? A compelling reason for making saccades is the need to explore a visual scene and to investigate several points of interest with high visual resolution and within a reasonable time. To achieve this, humans quickly shift their fovea toward visual information they detected in the periphery. The fovea is a small central area of the retina densely packed with cone photoreceptors providing the basis for a high-resolution percept at the center of the visual field. In the peripheral visual field, visual acuity and contrast sensitivity decrease (for a review on peripheral vision, see Rosenholtz, 2016; for a review on foveal and peripheral interactions, see Stewart et al., 2020). For some animals, which lack a fovea (receptor density is uniform across the retina), making saccades would have no effect considering that the resolution of the visual information would stay the same. The fact that those animals nonetheless make saccades leads to a second potential reason for why humans and other animals frequently interrupt fixations: avoiding perceptual fading due to unchanging retinal input over time, i.e., neuronal adaptation (Samonds et al., 2018). Neuronal adaptation is referred to as the decrease in spiking activity of neurons due to prolonged exposure to an unchanging, redundant input. Perceived contrast of an unchanging visual input will decrease with the reduced spiking activity in the neuronal population (Movshon and Lennie, 1979) and eventually, it will be erased from vision (perceptual fading, Troxler, 1804). While visual adaptation starts within tens of milliseconds (Müller et al., 1999), perceptual fading takes typically a few hundreds of milliseconds to several seconds (Riggs et al., 1953). However, not only a complete disappearance of visual input is detrimental for perception, the rapid reduction in perceived contrast (Foley and Boynton, 1993; Pavan et al., 2012) of relevant information is already something to avoid for efficient use of vision, which can be achieved by changing the retinal input frequently. The degree of change between inputs determines the degree of improved perceived contrast. Saccades are likely to achieve a large change between successive inputs when shifting gaze positions across distances outreaching the size of the fovea (Otero-Millan et al., 2013). However, Samonds et al. 2018 proposed that the distance that the eyes travel when a saccade is executed (saccade amplitude) is specifically optimized to maximize the change between presaccadic and postsaccadic inputs (i.e., to decorrelate both inputs). The authors proposed that preferred saccade amplitudes are based on the spatial properties of the visual scene (spatial frequency content) and the sizes of the areas from which neurons process information (receptive filed sizes).

Here, we ask the question whether this optimization of saccade amplitudes is necessary for perception. It might be that a saccade of any (larger) amplitude can induce a visual change that is sufficient to counteract neuronal adaptation. Adaptation may be rapid enough to affect perception within the range of typical fixation durations (Foley and Boynton, 1993; Pavan et al., 2012), but it is certainly weak given short fixation durations compared to prolonged fixations. This weak short-term adaptation might be easily counteracted by the dramatic changes that accompany saccades, for example by the motion streaks caused by the rapid movement of the eyes (Burr and Ross, 1982), which may be so disruptive that they are suppressed (to some extent probably actively) from conscious perception (for reviews, see Ross et al., 2001; Ibbotson and Krekelberg, 2011; Binda and Morrone, 2018). Indeed, the retinal input caused by a saccade-like motion has been shown to strongly alter spiking activity of retinal ganglion cells (e.g., Roska and Werblin, 2003; Idrees et al., 2020), and it is known that simple on-and-off flashing of a stimulus (attempting to imitate the effect of saccades) can delay its perceptual fading (Cornsweet, 1956; Bachy and Zaidi, 2014). However, it has been shown that unnaturally long adaptation (≥3 s) can survive a saccade and influence perception in humans (e.g., Melcher, 2005; 2007; Knapen et al., 2010; He et al., 2018) and that more natural short-term adaptation can attenuate contrast sensitivity and V1 activity across saccades in macaque monkeys (Gawne and Woods, 2003; Niemeyer and Paradiso, 2017). Hence, it is still an open question whether rapid adaptation can be strong enough to affect human perception across saccades as well.

## Results

**Figure 1:**
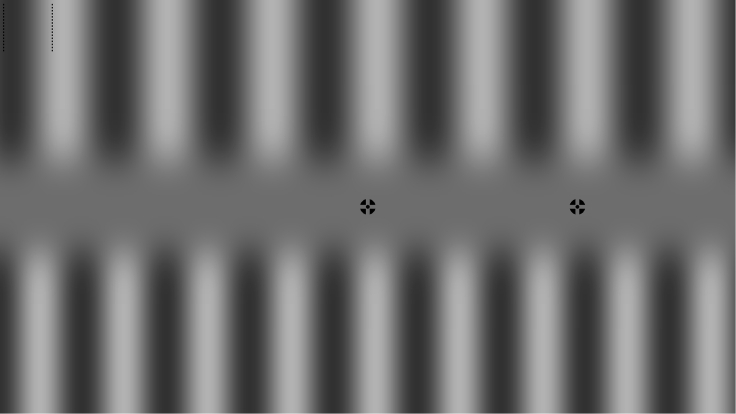
Demonstration of manipulation To reproduce appropriate spatial frequencies, one should view the image approximately at a distance of an arms lengths and adjust the size of the image such that one’s thumb placed on the image fits approximately in between the two dashed lines in the top left corner of the image. The described effect can be experienced by steadily fixating the central fixation stimulus for a few seconds, or (to achieve the largest possible effect) until the gratings begin to fade, and subsequently saccade towards the second fixation stimulus to the right. While fixating the second fixation stimulus, one should observe the top-half grating to be of lower contrast (correlated grating) than the bottom-half grating (anticorrelated grating). When the second fixation stimulus is fixated steadily, with low variance in gaze position, the effect can be observed for a long time.

**Figure 2:**
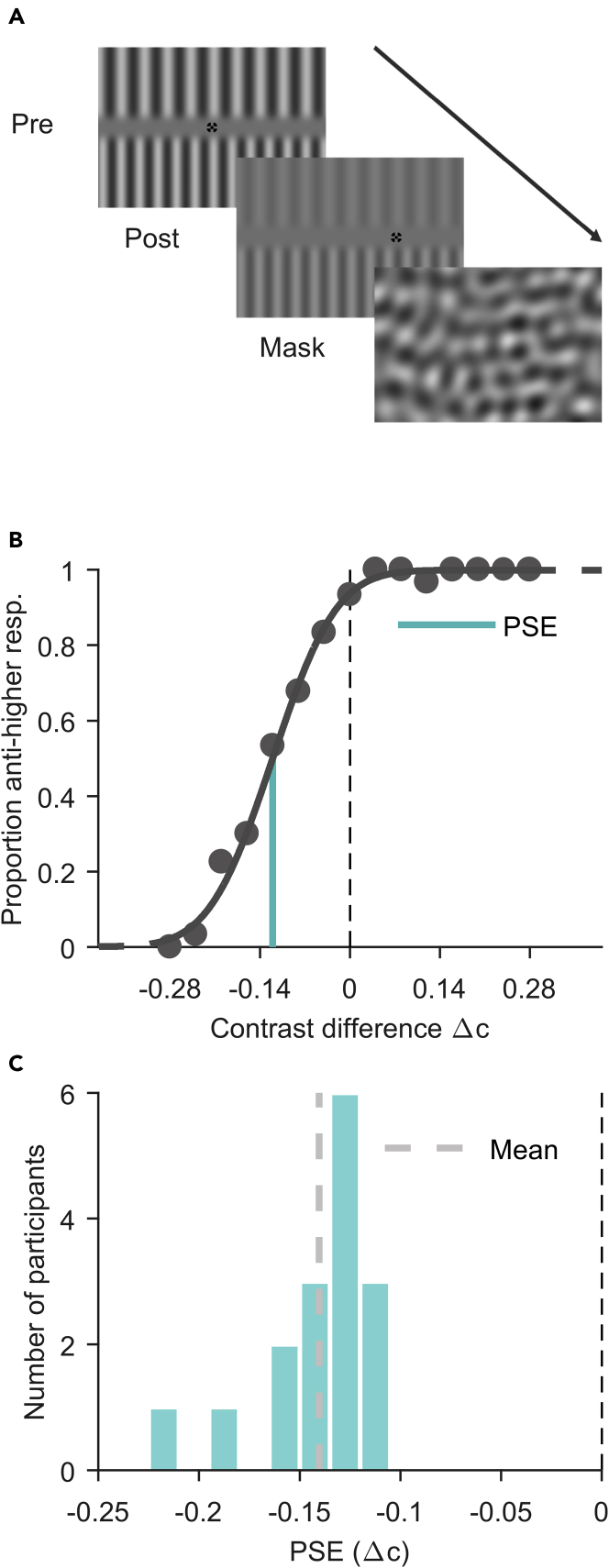
Trial procedure and main adaptation effect (Experiment 1) (A) The sequence of presaccadic (adaptation phase, until saccade onset), postsaccadic (test phase, 400ms duration) and mask (300ms duration) stimuli seen by a participant during a trial. The arrow indicates direction of time. Fixation stimuli are not drawn to scale. (B) Example data of one participant. The psychometric function fitted to proportion anticorrelated-grating-higher responses over contrast differences Δc illustrates a point-of-subjective-equality (PSE) shifted away from zero towards a negative contrast difference level (distance between dashed and turquoise line). A negative PSE indicates a higher perceived contrast of the anticorrelated grating. (C) Histogram of PSEs in contrast difference for all participants. The light-gray dashed line represents the mean PSE. The black dashed line represents a PSE of zero expected for unbiased responses.

With the present study, we address this question by applying a highly sensitive, novel variant of the contrast-cancellation method (Kelly and Martinez-Uriegas, 1993) in which perception of a maximal change (anticorrelated inputs) can be directly compared to that of a minimal/no change (correlated inputs) between inputs from before (presaccadic) and after a saccade (postsaccadic). Participants saw two adaptation gratings before and two test gratings after a horizontal saccade. They had to discriminate the contrast of the two postsaccadic gratings, one of which was correlated and one anticorrelated to their respective presaccadic grating due to the new gaze position (for a demonstration see Figure 1). Specifically, a presaccadic fixation stimulus was positioned at the center of the screen (during adaptation phase) and the position of the postsaccadic fixation stimulus (saccade target), which appeared after about 1.5 s, was shifted horizontally (left or right) from the center by twice the wavelength of one of the gratings (*correlated* grating, $F_{corr}=\;2$), which always corresponded to 1.5 or 2.5 times the wavelength of the other grating (*anticorrelated* grating, *F*_*anti*_ = 1.5 or 2.5). To avoid any undesired built-up of adaptation across trials, multiple properties of the stimuli were randomized across trials: the correlated and the anticorrelated grating were randomly assigned to the top and bottom location, the wavelength of the correlated grating and hence the eccentricity of the postsaccadic fixation stimulus was randomly jittered between ±5° and ±6.35° of visual angle (eccentricity: ±10° to ±12.5°) and the phase of the stimuli was chosen randomly. Provided that participants fixated both fixation stimuli accurately in a trial (we excluded trials in which fixation accuracy was not sufficient, for further details see STAR Methods), a participant's postsaccadic retinal input for one-half of the screen was replaced by an identical luminance pattern (white stays white and black stays black), and the other half was replaced by a luminance pattern of opposite phase (white becomes black and vice versa). After a subsequent mask stimulus (Figure 2A), participants had to indicate which of the two postsaccadic gratings had the higher contrast (upper or lower).

If the presaccadic fixation duration is sufficient for perceptual adaptation effects to emerge and outlive a saccade, contrast judgments comparing both halves of the screen during the postsaccadic test phase should reveal that a higher contrast of the correlated grating is needed to perceive the contrasts of both gratings as being equal. This would be indicated by a shift in the point of subjective equality (PSE, 50% “anticorrelated-grating-higher” judgments) to negative-contrast difference levels Δc ($\Delta c=c_{anti}-c_{corr}$), which we estimated from fitting psychometric functions to repeated contrast discrimination judgments on various levels of contrast differences (Figure 2B).

### Experiment 1: strong and robust adaptation

Average median saccade latency (time until saccade initiation after saccade target onset) over participants was 191 ± 39 ms (mean ± standard deviation) leading to a mean adaptation duration of 1705 ± 38 ms for Experiment 1. We found strong PSE shifts toward negative contrast differences (Figure 1C), indicating that the correlated grating needed to be higher in contrast by 0.14 ± 0.03 Δc, for both gratings to be perceived as equal (one-sided t test against zero: t(15) = −21.02, p < 0.0001). This means that we found robust perceptual adaptation effects, which persisted from one fixation to a next across a saccade.

### Experiment 2: rapidly emerging adaptation

**Figure 3:**
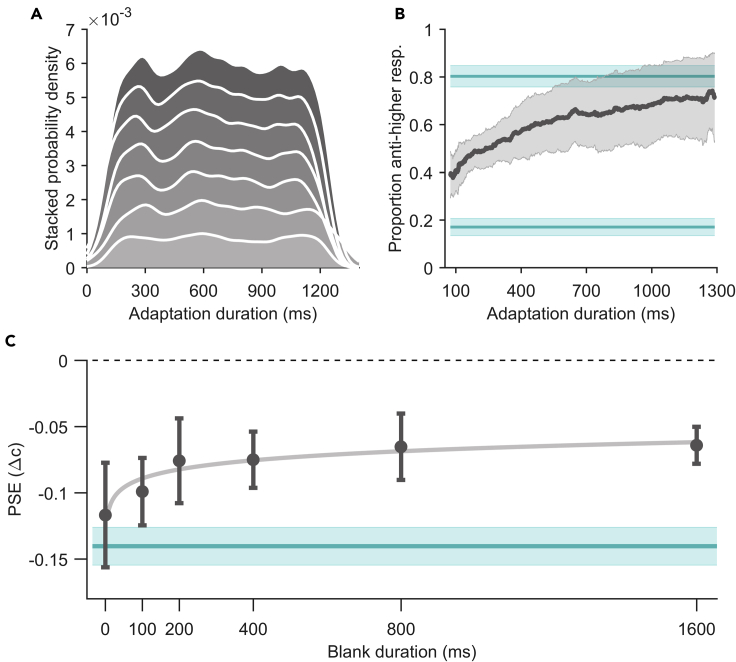
Results for build-up (Experiment 2) and decay (Experiment 3) of adaptation (A) Stacked probability density plot of number of valid trials over adaptation durations tested for seven participants in Experiment 2. Absolute number of trials can be found in Data Analysis. (B) Results testing the build-up of the adaptation effect over time in Experiment 2. Proportion of responses for perceiving the anticorrelated grating as of higher contrast over adaptation duration in milliseconds. The dark-gray line represents the mean proportion over participants and the light-gray area its 95%-confidence interval. The upper turquoise line represents the expected mean proportion of responses given the average PSE from Experiment 1. Likewise, the lower turquoise line represents the expected mean proportion of responses under the assumption of a PSE of zero (indicating no adaptation effect). The shaded area of both lines represents their 95%-confidence interval. (C) Results testing persistence of the adaptation effect over time in Experiment 3. PSE values in contrast difference over postsaccadic blank duration in milliseconds. Dots represent means across participants and error bars the 95%-confidence interval. The light-gray solid line represents the logarithmic function fitted to the mean data. The black dashed line at zero indicates the PSE value expected for no adaptation effect. The turquoise line (mean) and shaded area (95%-confidence interval) represent aggregated PSE values from Experiment 1.

In the second experiment, we went on to test the limits of this effect and whether it can emerge within more natural fixation durations of a few hundred milliseconds. Here, only one contrast difference level (−0.07) was tested and the adaptation duration varied in the range of 16 ms to 1,384 ms, which was partially dependent on the participant’s saccade latency in a given trial. For instance, if in a trial, we intended a 100-ms adaptation duration this would often be shorter than a typical saccade latency. Therefore, we estimated a participant's median saccade latency from previous trials and the adaptation stimulus would only be shown 100 ms before the predicted saccade onset (for further details, see STAR Methods). The average median saccade latency was 169 ± 23 ms and the aggregated number of valid trials over all 100ms bins (Figure 2A) averaged over participants was 26 ± 12. The proportion of responses indicating that the anticorrelated grating was perceived as of higher contrast (despite the fact that it had a physically lower contrast) increased with increasing adaptation durations (Figure 3B) and, importantly, was already significantly higher for the shortest adaptation durations tested (0.39 ± 0.09 for durations ≤100 ms) than the estimated baseline (0.17 ± 0.07 estimated from the fits in Experiment 1 when PSEs were set to zero; two-sample t test: t(21) = 6.55, p < 0.0001).

### Experiment 3: persistent adaptation

As the effect showed to be strong, robust, and rapidly emerging, we tested in a third experiment for how long it would persist when we inserted delays (blank-screen period) between adaptation and test phase of various lengths. The experiment was identical to the main experiment (Experiment 1), except the screen was blanked upon saccade detection for a certain period (100–1,600 ms) and some minor changes in the timing of the adaptation and the fixation stimulus (for further details, see STAR Methods). Average median saccade latency was 159 ± 24 ms and mean adaptation duration was 1,505 ± 3 ms for Experiment 3. PSE shifts decreased with blank durations and followed a logarithmic function starting at a PSE of −0.12 ± 0.04 Δc for no delay and reaching −0.06 ± 0.02 Δc for a delay of 1.6 s (Figure 3C). Since even the PSE for the longest delay was significantly below zero (one-sample t test against zero: t(6) = −11.24, p < 0.0001), these results indicate that the adaptation effect was also extremely persistent over time.

### Experiment 4: stronger adaptation around the center of gaze

**Figure 4:**
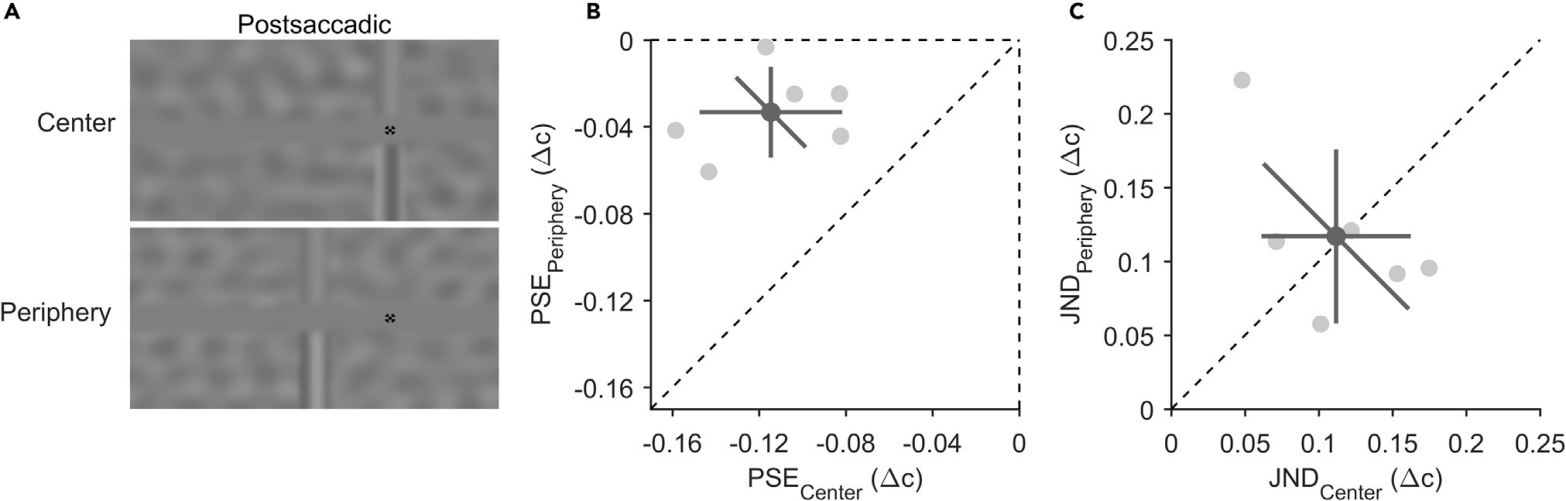
Test stimuli and results for contribution of visual field (Experiment 4) (A) Example postsaccadic test displays when the test-stimulus slice was presented around the center of gaze (upper panel) or to the periphery (lower panel). Fixation stimuli are not drawn to scale. (B) Scatter plot for all points of subjective equality (PSE) compared between the central condition (horizontal axis) and peripheral condition (vertical axis). Data points above the dashed vertical line indicate a stronger adaptation effect for when the test-stimulus slice was presented around the center of gaze. (C) Scatter plot for just-noticeable differences (JNDs) compared between the central condition (horizontal axis) and peripheral condition (vertical axis). Data points on the diagonal dashed line indicate that participants were equally precise in both conditions. (B and C) Light-gray dots represent individual participant data and the dark-gray dot indicates the overall mean. The error bars indicate 95%-confidence intervals within each condition (cardinal bars) or between conditions (oblique bar).

To investigate whether the observed adaptation effect was primarily driven by adaptation around the center of gaze or in the periphery, we tested a separate group of participants with a similar paradigm to Experiment 1, with the only difference being that the postsaccadic test stimuli were reduced to only a vertical slice of both gratings (a noise mask covered the rest of the gratings) positioned either around the center of gaze at the saccade target position (central condition) or in the periphery at the presaccadic fixation position (peripheral condition, Figure 4A). Average median saccade latency was 219 ± 64 ms for the central condition and 234 ± 80 ms for the peripheral condition leading to a mean adaptation duration of 1724 ± 62 ms for the central condition and 1742 ± 73 ms for the peripheral condition. Negative PSE shifts (Figure 3B) show an effect of adaptation around the center of gaze (−0.11 ± 0.03 Δc; t(5) = −8.96, p < 0.001) and in the periphery (−0.03 ± 0.02 Δc; t(5) = −4.09, p = 0.009), and the adaptation effect was significantly stronger around the center of gaze compared to the periphery (t(5) = −6.48, p = 0.001). This difference cannot be due to a lower discrimination performance in the periphery as JNDs (Figure 4C) were approximately equal for both conditions (t(5) = −0.14, p = 0.898). The results indicate that the PSE shifts from a central and peripheral part of the visual field almost perfectly add up to the PSE shift observed with a full test stimulus in Experiment 1 (−0.14 ± 0.03 Δc) and that a large portion of the adaptation effect is driven by the area around the center of gaze.

### Experiment 5: adaptation affects postsaccadic stimuli with high contrast

Finally, we tested if the adaptation affects perception also when the contrast of the postsaccadic stimulus is as high as that of the presaccadic stimulus. Experiment 5 was similar to Experiment 1 with the differences that the contrast of the adaptation stimulus was jittered across trials between 0.3 and 0.7, that the overall contrast of the test stimulus was equal to that of the adaptation stimulus, and that there was no mask following the test stimulus. Mean saccade latency and adaptation duration was 181 ± 16 ms and 1,692 ± 15 ms, respectively.

As the perceived luminance contrast of afterimages follows, but is usually weaker than, the luminance contrast of the adaptation stimulus (Kelly and Martinez-Uriegas, 1993), and given that an increment in contrast difference between gratings (due to the adaptation) is perceptually weaker when the contrast of the test stimulus is higher (Weber contrast), smaller adaptation effects can be expected with the design of this experiment. Accordingly, PSE shifts were smaller (mean PSE = −0.02 ± 0.01 Δc), but they were robust and significantly different from zero t(3) = −4.94, p = 0.008. This indicates that short-term adaptation can affect perception even when the postsaccadic contrast is high.

## Discussion

By comparing the perceived contrast of two postsaccadic gratings of which one was correlated and one was anticorrelated with the presaccadic retinal input, we found that short-term perceptual adaptation survives saccades, that it can build-up rapidly between saccades within the typical duration of fixations, and that it can last for more than one second. When we reduced postsaccadic test stimuli to either a central or peripheral part of the visual field, we found that the observed adaptation effect was predominantly driven by the visual field around the center of gaze position.

### Implications for natural fixation behavior

Given this time course for build-up and decay of the perceptual effect, we conclude that perceptual fading due to correlations between presaccadic and postsaccadic inputs can have dramatic consequences for vision and that neuronal activation or refresh due to retinal smear (Burr and Ross, 1982) or saccadic suppression of contrast sensitivity (for reviews, see Ross et al., 2001; Ibbotson and Krekelberg, 2011; Binda and Morrone, 2018) is not sufficient to washout short-term adaptation. Some authors argued that adaptation within natural fixation durations would be too weak or short-lived to affect perception (e.g., Poletti and Rucci, 2016). However, previous (Foley and Boynton, 1993; Pavan et al., 2012) and our perceptual results suggest that adaptation is not merely a challenge for prolonged fixations but also for the typical fixation durations of 200–300 ms that occur during natural exploration (Wilming et al., 2017).

Classic studies on the time course of neuronal adaptation (e.g., Müller et al., 1999) did not take into account the effect of fixational eye movements – such as microsaccades and ocular drift, which can delay perceptual fading by introducing small changes to the retinal input during fixation (Martinez-Conde et al., 2004). The role of fixational eye movements in counteracting perceptual fading is however strongly debated (for a review see Poletti and Rucci, 2016). Specifically in our study, their role might be negligible due to the low spatial frequencies of the gratings (Tulunay-Keesey, 1982). As microsaccade amplitudes are in the range of 0.05°–0.5° of visual angle (Poletti and Rucci, 2016), they are by definition not large enough to induce considerable changes to the retinal input given our low spatial frequency stimuli (minimal bar width = 2°). In general, small fixational eye movements should only decorrelate inputs on a correspondingly small spatial scale, i.e., in the high spatial frequency range, whereas large saccades decorrelate also in the low spatial frequency range. We have exclusively used low spatial frequencies (0.12–0.25 cyc/°) in our stimuli to make the intended manipulation work: full correlation or anticorrelation between retinal inputs across saccades could not be achieved if participants made saccadic landing errors, which they usually do but in a predictable range of 0.5°–1° of visual angle for larger horizontal saccades as used here (on average 11.25°; van Opstal and van Gisbergen, 1989). The lower spatial frequencies, i.e., the large bar widths in our gratings offer a larger tolerance for saccade landing errors and hence enabled us to reduce the expected number of invalid trials. While the low spatial frequencies made this manipulation feasible, low spatial frequencies also typically dominate natural scenes (Burton and Moorhead, 1987; Field, 1987; Ruderman and Bialek, 1994) and the combination of contrast and spatial frequencies we used in the adaptation stimulus is comparable to skies or grounds in natural-scene images (Frazor and Geisler, 2006; see also Figure S1). As natural stimuli nevertheless consist of a variety of spatial frequencies and orientations, a high degree of, but not necessarily, full correlation is likely to occur between consecutive fixations.

### Decorrelation of inputs as explicit purpose of saccades

It may still be discussed whether the perceptual refresh due to a large saccade is a coincidental by-product rather than based on accurate evaluation of spatial properties of a natural scene as suggested by Samonds et al., 2018 and that the latter would only apply if there is no superior goal that leads the saccade such as bringing a target of interest into the fovea. That counteracting perceptual fading is a by-product rather than a purpose has been proposed for fixational eye movements given that microsaccade or drift rates did not increase (rather decrease) with advancing perceptual fading (Cornsweet, 1956; Poletti and Rucci, 2010). The purpose of fixational eye movements may exclusively be increasing the precision of fixations (Cornsweet, 1956; Poletti and Rucci, 2010) and such enhancing the perception of fine spatial detail (e.g., Rucci et al., 2007; Rucci and Desbordes, 2003). Placing the fovea onto a region of interest might in turn be the behaviorally most relevant function for large saccades; but it is likely also a more modern function in evolutionary terms. For example, many fish make saccades despite not having a fovea to place a target at for detailed inspection and do not show saccade behavior specific to target inspection (for a review, see Land, 2019). In addition, Samonds et al., 2018 could show that saccade amplitudes in afoveate mice scale with their visual acuity (upper limit of spatial frequency range obtainable) during passive viewing. Importantly, human saccade and fixation behavior in passive viewing depend on spatial properties of the scene consistent with the decorrelation hypothesis: Groner et al., 2008 demonstrated that human saccade amplitudes become larger when inspecting natural images with increased low spatial frequency content compared to high spatial frequency content. In addition, Mostofi et al., 2020 showed that saccades reduce redundant luminance information (i.e., the power of low spatial frequencies) obtainable from natural scenes over time when the scene is passively explored. Krieger et al., 2000 could show that regions with higher spatial variance (higher potential to lead to decorrelation) were more likely to be fixated. In addition, patches of decorrelated input in the visual field are likely to attract gaze orienting (Ludwig et al., 2012). In conclusion, perceptual refresh may not be the primary function of saccades in daily vision but likely one in passive viewing and a more deep-rooted function in general, as it is based on fundamental neuronal properties.

### Neuronal locus of the perceptual adaptation effect

Adaptation is a ubiquitous neuronal property inherent to every processing level in the visual hierarchy (for reviews, see Kohn, 2007; Webster, 2015) and rapid adaptation in lower-level visual areas can be passed on to higher-level visual areas and affect behavior (Jin and Glickfeld, 2020). As the adaptation effect we measured is retinotopic and specific to the phase of the gratings, the neuronal locus for this effect should not go beyond simple cells (Hubel and Wiesel, 1962), in the primary visual cortex (V1) because processing is phase-invariant in complex cells and at higher processing levels. At the lower end, it could have its origin as early as in the bleaching of cone photoreceptors. Negative afterimages resulting from mid-photopic stimulation (such as here) should, however, have their origin in the adaptation of retinal ganglion cells (Zaidi et al., 2012). Given that we found a stronger adaptation effect in the central visual field compared to the periphery and that an adapted state itself does not remap across saccades (He et al., 2018), one could conclude that the observed proportional contributions to the adaptation effect are related to the varying density of ganglion cells across the visual field, which decreases with increasing eccentricity (Wässle et al., 1990).

### Conclusions

We show that luminance adaptation can be strong, rapid, and persistent enough to attenuate contrast perception within natural fixation durations and that this effect can outlast a large saccade despite the strong disruption of visual input. Our findings indicate that attenuation of postsaccadic contrast can indeed be a relevant issue for human perception and that therefore, decorrelation of retinal inputs might be a plausible objective to constrain the range of potential saccade amplitudes (Samonds et al., 2018).

### Limitations of the study

To investigate the possibility of short-term adaptation to affect perception, we aimed to make the effect as visible as possible by 1) letting participants compare a correlated and an anticorrelated grating and 2) by using a lower mean contrast of the postsaccadic test stimuli. Regarding the first point, many previous studies either investigated adaptation effects on the perception of a single correlated stimulus (e.g., Kelly and Martinez-Uriegas, 1993) or on the removal of the adapted stimulus to investigate the perception of afterimages (e.g., Tulunay-Keesey, 1982). In comparison to these methods, our method might give us an upper bound of the effect as we did not measure the reduction (correlation) and increase (decorrelation) in perceived contrast separately but measured the difference between the perceived contrast of correlated and anticorrelated stimuli. Regarding the second point, we conducted Experiment 5 where the mean contrast of the test stimuli was as high as the contrast of the adaptation stimuli. The effect was smaller but nevertheless robust and significantly different from zero. However, also in this version, we used an adaptation duration that is brief compared to most adaptation studies but still exceeding typical fixation durations. The effect is likely to diminish with a reduced adaptation duration as indicated by the results of Experiment 2. While it is hence arguable that an adaptation effect due to correlations between presaccadic and postsaccadic inputs might be noticeable under more natural conditions, we see our study as proof of principle and are convinced that even a very small attenuation of perceived contrast may matter to the visual system.

## STAR★Methods

### Key resources table

**Table:** 

REAGENT or RESOURCE	SOURCE	IDENTIFIER
**Deposited data**		

Eye-movement data and perceptual data	Zenodo.org	https://doi.org/10.5281/zenodo.4568210
Software and Algorithms		
MATLAB R2017a	Mathworks, Natick, MA, USA	https://www.mathworks.com/products/matlab.html, RRID:SCR_001622
Psychtoolbox 3, including Eyelinktoolbox	Brainard, 1997; Pelli, 1997; Cornelissen et al., 2002	http://psychtoolbox.org/, RRID:SCR_002881

**Other**		

Eyelink 1000+ eye tracker	SR Research Ltd., Ontario, Canada	https://www.sr-research.com/products/eyelink-1000-plus/, RRID:SCR_009602
VIEWPixx monitor	VPixx Technologies Inc, Saint-Bruno, QC Canada	http://vpixx.com/products/viewpixx/, RRID:SCR_013271

### Resource availability

#### Lead contact

Further information and requests for resources should be directed to and will be fulfilled by the lead contact, Alexander C. Schütz (a.schuetz@uni-marburg.de).

#### Materials availability

This study did not generate new specimens or materials. All images are included in the text and Supporting Information.

### Experimental model and subject details

In Experiment 1, we tested twenty participants who were unaware of the purpose of the study. Three of them discontinued the experiment before a sufficient number of trials for analysis was completed. Another participant was excluded from further analysis after data inspection revealed that the participant misinterpreted the experimental task (gratings discriminated by spatial frequency instead of contrast). The data of sixteen participants (12 female, 4 male; mean age = 22 years, range = 18–27 years) was used for analysis. In Experiments 2 and 3, six participants who were unaware of the purpose of the study and one author (CH) were tested and included into analysis (5 female, 2 male; mean age = 25 years, range = 22–33 years). In Experiment 4, we tested and included six participants who were unaware of the purpose of the study (5 female, 1 male; mean age = 23 years, range = 22–24 years). In Experiment 5, three participants unaware of the purpose of the experiment and one of the authors (4 female; mean age = 25 years, range = 22–29 years) were tested and included into analysis. All participants were either students or associates of Marburg University, had normal or corrected-to-normal vision, and gave informed consent prior participation. The study was conducted in accordance with the principles of the Declaration of Helsinki 1964 and authorized by the local ethics committee of the psychology department at Marburg University (proposal number 2015-35k).

### Method details

#### Stimuli

The horizontal grey bar separating the upper and lower luminance gratings in adaptation- and test phase was created by applying a one-dimensional, generalised gaussian (inversed) window (scale α = 2.1° of visual angle, shape β = 3) elongated to the length of the display. By this, the edges of the grey bar blend into the gratings and the center of the bar is fully oblique. Pre- and postsaccadic fixation stimuli were black and a combination of a bull's-eye and crosshair (Thaler et al., 2013) with a diameter of 0.6°. The wavelength of the correlated grating was chosen randomly between 5° and 6.25° (spatial frequency: 0.16 to 0.2 cyc/°). The wavelength of the anticorrelated grating ($\lambda_{anti})\;$fulfils the equation $\lambda_{anti}=\frac{\left(\lambda_{corr}*2\right)}{F_{anti}}$, with the factor $F_{anti}$ being either 1.5 or 2.5. Hence, the wavelength of the anticorrelated grating varied between 4° and 8.3° (spatial frequency: 0.12 to 0.25 cyc/°). The common phase of the two gratings was jittered randomly between −0.5 π and 0.5 π. While the spatial properties of the gratings remained the same for adaptation and test phase, contrast was differing. The contrast of the adaptation stimulus (both gratings before saccade) was 0.5. The constant mean contrast level of the test stimulus (both gratings after saccade) was 0.15 (the mean contrasts were different in Experiment 5, see design). A mask stimulus following the test stimulus covered the entire screen with Gaussian-filtered white noise with an average spatial frequency of 0.18 ± 0.02 cyc/° and a contrast of 0.7. The test stimuli of Experiment 4 (Figure 3A) differed from the test stimuli of Experiments 1–3 by the following: both gratings were covered by a mask stimulus that included a vertical generalised gaussian window (α = 2.1°, β = 5). This window uncovered a slice of the gratings and its center was positioned either at the postsaccadic target location (central condition) or at the screen center (peripheral condition).

#### Equipment

Stimuli were displayed on a VIEWPixx monitor in M16 mode (greyscale) at a 1920 × 1080-pixel resolution and a 120-Hz refresh rate. The display had a size of 51.5 × 29 cm and was viewed at a distance of 60 cm. Luminance was 0.21 cd/m^2^ for black, 105.70 cd/m^2^ for white, and 58.33 cd/m^2^ for grey pixels. Eye movements were recorded with a desktop-mounted EyeLink 1000 (SR Research Ltd., Ontario, Canada) with a sampling rate of 1,000 Hz. Experimental software and analysis were written in MATLAB R2017a (Mathworks, Natick, MA, USA) using the Psychophysics Toolbox (Brainard, 1997; Pelli, 1997) for stimulus display and the Eyelink Toolbox (Cornelissen et al., 2002) for eye tracker operation. Participants responded using a standard keyboard. Participant's head position was stabilized using a forehead- and chinrest.

#### Design

For Experiments 1 and 4, the contrast difference between the anticorrelated- and the correlated grating took on values from minus and plus 0.28 in steps of 0.04, with negative values indicating a higher contrast of the correlated grating. In Experiment 2, only one contrast difference of −0.07 was tested. In Experiment 3, tested contrast differences ranged between −0.3 and 0.2 in steps of 0.05. The method of constant stimuli (Fechner, 1860) was used in Experiments 1, 3, 4, and 5 to obtain psychometric functions. Contrast differences, the position of the correlated- and anticorrelated gratings (upper or lower half) and the value of $F_{anti}$, and the conditions in Experiment 2 (intended adaptation duration: 100–1,200 ms in 100ms steps) and 3 (postsaccadic blank duration: 0, 100, 200, 400, 800, and 1,600 ms), were counterbalanced and trial order was randomised. In Experiment 4, the two conditions (location of slice: center, periphery) were blocked (block order was counterbalanced across participants) and within each block contrast differences, grating position, and value of F were counterbalanced and trial order was randomised. Each factor combination was measured at least 8 times in Experiments 1 and 3 resulting in 480 and 528 trials respectively, four times in Experiment 2 resulting in 384 trials, and two times in Experiment 4, resulting in 120 trials per block. Experiment 5 was similar to Experiment 1 with the differences that the contrast of the adaptation stimulus was randomly jittered between 0.3 and 0.7 across trials, that the overall contrast of the test stimulus was equal to that of the adaptation stimulus, and that there was no mask following the test stimulus. Contrast difference levels tested in Experiment 5 were −0.4, −0.3, −0.2, −0.15, −0.1, −0.05, 0, 0.05, 0.1, 0.15, 0.2, 0.3, 0.4.

#### Eye-tracker calibration

The eye tracker was calibrated using the participant's right eye, for 9 locations (marked by a fixation stimulus) in a grid array with one location at the center of the screen and the remaining with an eccentricity of 13° of visual angle on the horizontal and/or 5° on the vertical axis. The experimenter confirmed gaze position at each location manually, while ensuring that each difference between computed gaze position and stimulus location was below 0.5° and below 0.35° on average during validation. The calibration procedure was conducted before the start of each experiment, and after every 100 trials. In addition to the calibration procedure, at the start of each trial a drift correction was implemented, that was manually confirmed by the participant using the space bar on the keyboard.

#### Procedure

Participants started each trial by pressing the space bar while fixating a central fixation. In Experiments 1, 4, and 5 the adaptation stimuli were displayed upon trial initiation and the saccade target (also postsaccadic fixation stimulus) was added at an eccentricity of twice the wavelength of the correlated grating to the left or right (between ±10° and ±12.5°) after a duration chosen at random between 1.4 and 1.6 s. The central fixation stimulus disappeared after an additional 200 ms or when a saccade was detected (overlap paradigm, Saslow, 1967). A saccade was detected when the recorded gaze position exceeded 2° of visual angle in respect to screen center. Upon saccade detection, the adaptation stimulus was replaced by the test stimulus, which was 400ms later replaced by a mask (except Experiment 5, which did not have a mask but the screen turned grey after 400 ms). After an additional 300 ms the screen turned grey, which prompted the participants to respond by pressing the up-arrow key for reporting to have perceived the upper half as of higher contrast or the down-arrow key for reporting to have perceived the lower half as of higher contrast. Auditory feedback was given after each trial for inaccurate fixation- or saccade behaviour towards the pre- or postsaccadic fixation stimulus position.

The procedure of Experiment 2 differed from the procedure of Experiment 1 by the following: the central fixation stimulus disappeared upon onset of the saccade target (no overlap paradigm, note that the adaptation stimulus i.e., the gratings, are distinct from the fixation stimuli) and the onset of the saccade target was fixed in time to 1.2 s after trial initiation. To enable very short adaptation durations (e.g., 100 ms), and assuming that adaptation continues during saccade preparation, the saccade latency of the participant was estimated by taking the median saccade latency of the previous 20 trials available. The saccade-latency estimate for the first trial was set to 190 ms. If the estimated saccade latency on a given trial was equal to the intended adaptation duration, saccade target and adaptation stimulus were displayed simultaneously (preceded by 1.2 s of grey screen and the central fixation stimulus). If the estimated saccade latency was longer than the intended adaptation duration, the onset of the saccade target was followed by the onset of the adaptation stimulus by the respective time difference. The order was reversed when the estimated saccade latency was shorter than the intended adaptation duration i.e., the onset of the adaptation stimulus preceded the onset of the saccade target. As in Experiment 1, the adaptation stimulus was replaced by the test stimulus upon saccade detection.

Saccade latency in Experiment 3 was estimated in the same way as in Experiment 2. One purpose here was to ensure a relatively constant adaptation duration within and across participants of 1.5 s. That is, the onset of the adaptation stimulus after trial initiation was delayed by the estimated saccade latency and saccade target onset was set to 1.5 s after trial initiation. The central fixation stimulus disappeared upon saccade target onset (no overlap paradigm). The adaptation stimulus disappeared upon saccade detection and the subsequent onset of the test stimulus was delayed by the intended blank duration. In all remaining aspects the procedure of Experiment 3 was similar to that of Experiment 1.

#### Trial exclusions

Single trials were excluded from analysis based on participant's fixation accuracy in adaptation- and test phase as the position of gaze with respect to the gratings was crucial to our manipulation. For all experiments, we excluded trials that contained blinks in the time between trial initiation and mask onset, trials in which the switch between adaptation- and test stimuli was not achieved in the time of the saccade (e.g. due to small, consecutive saccades instead of one large saccade), and trials in which the standard deviation of horizontal gaze positions sampled between adaptation-stimulus onset and saccade onset was above 0.5°. The latter was to ensure a stable gaze position during the adaptation phase. Horizontal saccade amplitude led to trial exclusion based on the ratio of saccade amplitude and correlated-grating wavelength ($F_{corr}=Ampl/\lambda_{corr}$) and the absolute difference to its nearest natural number. A difference of 0.5 is the worst possible outcome in respect to our manipulation, as this would mean that the participant's saccade amplitude inverted the characteristics of the two gratings (correlated became anticorrelated grating and vice versa). A difference of zero corresponds to the best-case scenario. We excluded all trials in which the difference was equal to or above the boundary between best and worst case of 0.25.

In addition to the exclusion criteria common to all experiments, we excluded trials of Experiment 1, 4 and 5 in which the saccade latency was above 600 ms (<1% of trials), and trials of Experiment 3 in which the adaptation duration deviated more than 100 ms from the intended adaptation duration of 1.5 s (6 ± 5% of trials). Total amount of trials excluded was 9 ± 9% for Experiment 1, 9 ± 5% for Experiment 2, 7 ± 6% for Experiment 3, 2 ± 3% (central condition) and 8 ± 12% (peripheral condition) for Experiment 4, and 19 ± 18% of trials for Experiment 5.

### Quantification and statistical analysis

#### Eye-movement data analysis

For eye-movement data analysis saccades were detected offline using the EyeLink 1000 algorithm (velocity threshold = 22°/s, acceleration threshold = 3800°/s^2^). Saccade onsets were defined as the first sample after saccade-target onset in which a saccade was detected; likewise, saccade offsets were defined as the last sample after saccade onset in which a saccade was detected. Presaccadic fixation position was defined as the mean of all gaze positions sampled between adaptation-stimulus onset and saccade onset. Postsaccadic fixation position was defined as the mean of gaze positions sampled between saccade offset and mask onset, during the time the test stimulus was presented. Saccade amplitude was defined as the difference between post- and presaccadic fixation position. Saccade latency was defined as the time (resolution of 1 ms) between saccade-target onset and saccade onset. Adaptation duration was defined as the time between adaptation-stimuli onset and saccade onset.

#### Response data analysis

To obtain psychometric functions for Experiments 1, 3, 4, and 5, perceptual choices were sorted by contrast-higher responses for the anticorrelated grating (anticorrelated-higher responses) and their proportion was calculated for each contrast-difference level tested. A cumulative Gaussian was fitted to the data using psignifit 4.0 toolbox (Schütt et al., 2016). The point of subjective equality (PSE) was estimated as the level of contrast difference corresponding to 50% anticorrelated-higher responses. A negative PSE indicates a perceptual bias for perceiving the correlated grating of lower contrast and the anticorrelated grating of higher contrast. The just-noticeable difference (JND) was defined as the standard deviation of the cumulative Gaussian, with a lower JND indicating higher precision of the contrast discrimination.

For Experiment 2, proportions anticorrelated-higher responses (for the one contrast-difference level tested) were calculated as a running average over all adaptation durations that resulted from the procedure. The adaptation durations reached values between 16 and 1,384 ms and the average number of trials per 100ms bin (range 0–1,400 ms) was 26 ± 2 across participants. The running average was calculated starting from 0 ms in 300ms bins and 1ms steps ending at 1,400 ms. The very first bin was only half the bin size (150) and it was increased by 1 until it the full bin size of 300 was reached; until then, every bin's starting point remained at zero milliseconds (0–150 ms, 0–151 ms, …, 0–300 ms). The same procedure but reversed was applied for the end of the range (1,100–1,400 ms, 1,101–1,400 ms, …, 1,250–1,400 ms). The proportion of anticorrelated-higher responses was assigned to the mean of each bin.

For Experiment 3 we fitted a natural logarithm to the PSEs for each participant and for mean PSEs using the implemented log-function in MATLAB and two free parameters A and B following the equation y=A∗log(x)+B.

All statistical test were *t*-tests, made using MATLAB R2017a (Mathworks, Natick, MA, USA) software, and the alpha value was set to 0.05. Statistical details and as well as relevant means and standard deviations in the format Mean ± SD, can be found in the Results section.

#### Additional results for Experiments 1 and 3

From the psychometric functions fitted to the response data of Experiments 1 and 3 we also estimated the just-noticeable difference (JND) indicating the precision of a participant in judging the contrast difference between the postsaccadic gratings. Mean JND over participants of Experiment 1 was 0.077 ± 0.023 Δc (mean ± standard deviation) and for participants of Experiment 3 mean JNDs were 0.057 ± 0.025, 0.041 ± 0.019, 0.041 ± 0.014, 0.046 ± 0.018, 0.046 ± 0.009, 0.048 ± 0.012 Δc for each blank duration (0 ms, 100 ms, 200 ms, 400 ms, 800 ms, 1,600 ms), respectively.

#### Exploratory results for Experiment 1

As an exploratory analysis of Experiment 1, we sorted trials for when $F_{anti}$ equalled either 2.5, or 1.5. With $F_{corr}=2$, the factor for the anticorrelated grating either led to a higher spatial frequency of the anticorrelated grating (when $F_{anti}=\;$2.5), or a lower spatial frequency of the anticorrelated grating (when $F_{anti}=\;$1.5) compared to the correlated grating. Psychometric functions were fitted to each of the datasets for each participant and the PSEs were estimated (Figure S2A). A one-sample, paired t test revealed a significant difference of PSEs for spatial frequency, t(15) = 4.38, p < 0.001, while JNDs did not differ (t(15) = 1.72, p = 0.107). The result indicates that the observed adaptation effect was stronger when the correlated grating had a lower spatial frequency than the anticorrelated grating. A possible explanation could be that lower spatial frequencies come with a larger range of possible postsaccadic fixation positions in which the retinal input remains unchanged, making our manipulation more tolerant.

In our paradigm, visual stimulation was specific for the lower and the upper visual field, which offered the opportunity to investigate perceptual effects with regard to visual-field location. Perceptual differences between the upper and lower visual field often reveal superiority of the lower visual field, which can be traced back to topographical asymmetries in the retina (for reviews, see Skrandies, 1987; Karim and Kojima, 2010). However, this relationship seems to be reversed in the superior colliculus, where the upper visual field seems to have priority (Hafed and Chen, 2016). Similar to the aforementioned analysis, we investigated the effect location of the gratings (Figure S2B). PSEs for when the correlated grating filled the lower half of the screen were significantly more negative than when the correlated grating was at the upper half of the screen, t(15) = 3.04, p = 0.008, while JNDs did not differ (t(15) = 1.34, p = 0.202). This visual field difference could be explained in two different ways: first, it might reflect a bias in contrast perception such that perceived contrast is lower in the lower compared to the upper visual field. A previous study reported the opposite effect, with higher perceived contrast in the lower compared to the upper visual field along the vertical meridian (Fuller et al., 2008). However, the effect found by Fuller et al. (2008) diminished with decreasing eccentricity (2 vs. 4 cyc/°), which indicates that this perceptual asymmetry might be strongly reduced with the low spatial frequency stimuli of our study (0.12–0.25 cyc/°). Second, it might reflect an asymmetry in adaptation, such that adaptation is faster or stronger in the lower than the upper visual field, which may be evidenced by the on average higher ganglion-cell density in the lower visual field (for review see Skrandies, 1987). This asymmetry can, however, only be a cause when the increase in perceived contrast due to a refresh (anticorrelated grating) is not proportional to the reduction in perceived contrast due to a cancelation (correlated grating). This possibility remains to be tested in the future.

## Data Availability

Eye-movement data and perceptual data have been deposited at Zenodo and are publicly available as of the date of publication. DOIs are listed in the key resources table. This paper does not report original code. Any additional information required to reanalyze the data reported in this paper is available from the lead contact upon request.
