# Supplementary material for: Rapid visual adaptation persists across saccades
Source: iScience. 2021 Aug 16;24(9):102986. doi: 10.1016/j.isci.2021.102986 (PMC8403744; doi:10.1016/j.isci.2021.102986)
Supplement: Document S1. Figures S1 and S2 [file mmc1.pdf]

iScience, Volume *24*

Supplemental information

Rapid visual adaptation persists across saccades

Carolin Hübner and Alexander C. Schütz

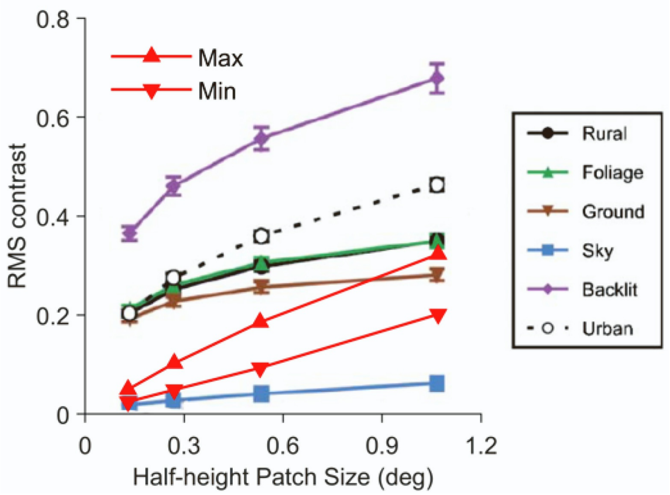

Figure S1. Comparison of RMS contrast in the adaptation stimuli to RMS contrast in natural scenes from Frazor & Geisler (2006), Related to Figure 2. *RMS contrast over patch size in degrees of visual angle. The legend on the right indicates the image region categories for the data reported by Frazor & Geisler (2006). The legend at the top left corner indicates the data we have added to the plot. Max stands for an adaptation stimulus with the highest spatial frequency that was show in our experiments (0.25 cpd) and Min stands for an adaptation stimulus with the lowest spatial frequency that was show (0.12 cpd). Local contrast increases with patch sizes for the adaptation stimuli as well as for the natural-image categories. Since the adaptation stimulus is entirely composed of low spatial frequencies, its RMS contrast is particularly low for small patch sizes. Overall, the RMS contrast of the adaptation stimuli lies within the RMS-contrast range for natural stimuli; specifically, their contrasts are comparable to that of image parts corresponding to skies or grounds.*

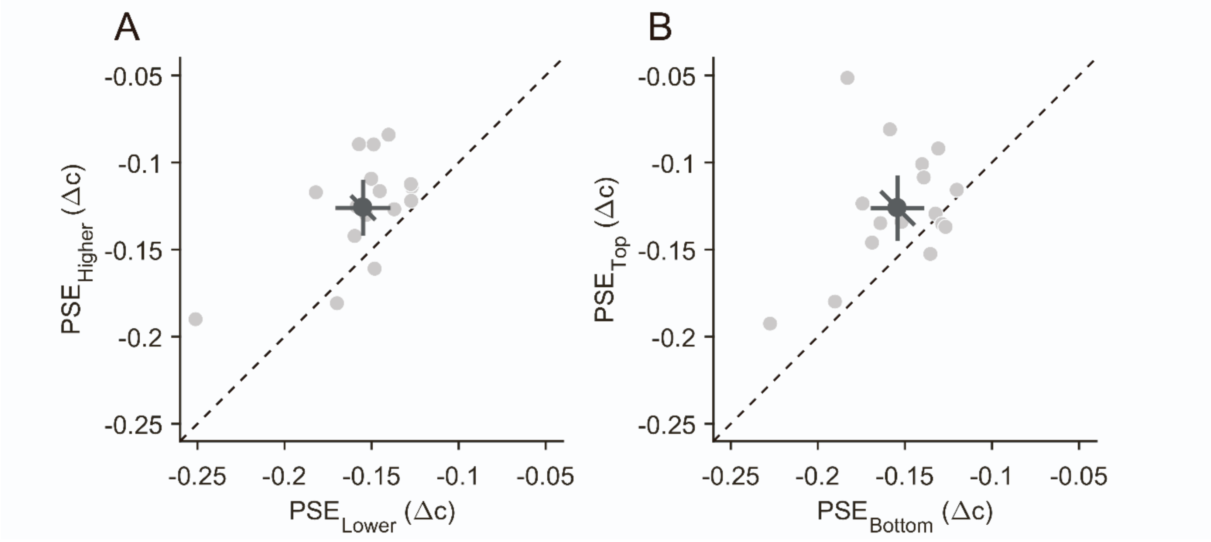

Figure S2. Exploratory results of Experiment 1, Related to Figure 2. *Individual PSE values (light-grey dots) and mean PSE (dark-grey dot) with 95% confidence intervals (grey bars) in units of contrast difference Δc between gratings.* ***(A)*** *Comparison of trials in which the correlated grating had a lower spatial frequency than the anticorrelated grating (horizontal axis) to trials in which the correlated grating had a higher spatial frequency (vertical axis).* ***(B)*** *Comparison of trials in which the correlated grating was at the bottom half of the screen (horizontal axis) to trials in which it was at the top half of the screen (vertical axis). Data points above the diagonal indicate a larger adaptation effect for the condition on the horizontal axis.*

References

Frazor, R. A., & Geisler, W. S. (2006). Local luminance and contrast in natural images. *Vision Research*, *46*(10), 1585–1598. https://doi.org/10.1016/j.visres.2005.06.038
